# Disease variants in genomes of 44 centenarians

**DOI:** 10.1002/mgg3.86

**Published:** 2014-06-15

**Authors:** Yun Freudenberg-Hua, Jan Freudenberg, Vladimir Vacic, Avinash Abhyankar, Anne-Katrin Emde, Danny Ben-Avraham, Nir Barzilai, Dayna Oschwald, Erika Christen, Jeremy Koppel, Blaine Greenwald, Robert B Darnell, Soren Germer, Gil Atzmon, Peter Davies

**Affiliations:** 1 The Litwin-Zucker Research Center for the Study of Alzheimer's Disease and Memory Disorders, The Feinstein Institute for Medical Research, North Shore-LIJManhasset, New York, 11030; 2 Division of Geriatric Psychiatry, Zucker Hillside Hospital, North Shore-LIJGlen Oaks, New York, 11040; 3 Robert S. Boas Center for Genomics and Human Genetics, The Feinstein Institute for Medical Research, North Shore-LIJManhasset, New York, 11030; 4 New York Genome Center101 Avenue of the Americas, New York, New York, 10013; 5 Institute for Aging Research Departments of Medicine and Genetics, Albert Einstein College of Medicine1300 Morris Park Avenue, Bronx, New York, 10461; 6 Department of Molecular Neuro-Oncology, Howard Hughes Medical Institute, The Rockefeller University1230 York Avenue, New York, New York, 10065

**Keywords:** Aging, Ashkenazi, centenarian, disease gene, incidental finding, whole genome sequencing

## Abstract

To identify previously reported disease mutations that are compatible with extraordinary longevity, we screened the coding regions of the genomes of 44 Ashkenazi Jewish centenarians. Individual genome sequences were generated with 30× coverage on the Illumina HiSeq 2000 and single-nucleotide variants were called with the genome analysis toolkit (GATK). We identified 130 coding variants that were annotated as “pathogenic” or “likely pathogenic” based on the ClinVar database and that are infrequent in the general population. These variants were previously reported to cause a wide range of degenerative, neoplastic, and cardiac diseases with autosomal dominant, autosomal recessive, and X-linked inheritance. Several of these variants are located in genes that harbor actionable incidental findings, according to the recommendations of the American College of Medical Genetics. In addition, we found risk variants for late-onset neurodegenerative diseases, such as the *APOE ε4* allele that was even present in a homozygous state in one centenarian who did not develop Alzheimer's disease. Our data demonstrate that the incidental finding of certain reported disease variants in an individual genome may not preclude an extraordinarily long life. When the observed variants are encountered in the context of clinical sequencing, it is thus important to exercise caution in justifying clinical decisions. In genome sequences of 44 Ashkenazi centenarians, we identified many coding variants that were annotated as “pathogenic” or “likely pathogenic” based on the ClinVar database. Our data demonstrate that the incidental finding of certain reported disease variants in an individual genome may not preclude an extraordinarily long life. When the observed variants are encountered in the context of clinical sequencing, it is thus important to exercise caution in justifying clinical decisions.

## Introduction

Human genetic studies have linked many variants to human diseases or nondisease phenotypes. How to handle the incidental finding of a disease variant is a topic of current discussion (Green et al. 2013a; Klitzman et al. 2013). Incidental findings often occur, when genome sequencing data are screened for disease-causing variants that are recorded in databases such as Online Mendelian Inheritance in Man (OMIM) (Hamosh et al. 2005) or, more recently, ClinVar (Landrum et al. 2014) and the Genome Wide Association Studies (GWAS) catalog (Hindorff et al. 2009). However, the penetrance of these recorded variants spans a broad spectrum, ranging from complete penetrance for a set of monogenic mutations to the very small effect sizes of many GWAS hits. While it is widely known that most GWAS hits have only limited clinical prognostic relevance, the penetrance and prognostic value of many previously reported monogenic mutations is less clear. Recently, a significant percentage of such putative “disease mutations” were claimed to be of questionable pathogenicity (Cassa et al. 2013; Dorschner et al. 2013; Flannick et al. 2013; Kenna et al. 2013; Riggs et al. 2013). Accordingly, it is an important aim of current human genetic research to systematically assess the clinical significance of genetic variants (Duzkale et al. 2013). As one way to identify those mutations that may require reinterpretation, we use a sample of individuals with exceptional longevity to identify reported disease mutations for which the pathogenicity status should be preferentially reevaluated.

If a mutation for a dominant late-onset disease is encountered in the genome of a younger individual, a reduced penetrance is difficult to assign without longitudinal follow-up. In addition to the uncertain significance of many reported mutations for dominant diseases, the traditional assumption that heterozygous carriers for recessive disease alleles are unaffected (Nussbaum et al. 2007) may not hold true. For instance, Gaucher's disease alleles in the *GBA* gene have been found to strongly increase the risk of developing Parkinson's disease (Sidransky et al. 2009) as well as dementia with Lewy bodies (Nalls et al. 2013). Similarly, heterozygous carriers for autosomal recessive disease alleles for Alport syndromes have increased risk of renal failure (Temme et al. 2012). Therefore, different lines of evidence need to be integrated to assign a clinical significance score to each variant. For mutations previously linked to dominant diseases that negatively affect life expectancy, their observation in centenarian genomes may be viewed as evidence for unclear pathogenicity or at least incomplete penetrance. To a certain extent, reduced penetrance could result from a variety of protective genetic, epigenetic, environmental, and random factors (Cooper et al. 2013) or other buffering mechanisms (Bergman et al. 2007).

In the present study, we search the coding regions of 44 Ashkenazi Jewish (AJ) centenarians for variants that were previously reported as causal mutations for medically relevant phenotypes. To this end, we use the recently established and publicly accessible ClinVar database (Riggs et al. 2013) which not only provides highly structured data access but also includes community-based data curation (Landrum et al. 2014). We chose ClinVar because of the quality of its content. Our study is meant to be part of the community effort to further improve its quality, which is eased by its free availability. Such databases are gaining in importance, because the increased utilization of next-generation sequencing technology magnifies the challenge to interpret genetic testing results (Lyon and Wang 2012; Lohn et al. 2013; Manolio et al. 2013; Rehm et al. 2013). For example, in August 2013 the ClinVar database recorded 14,746 variants classified as pathogenic and 1672 as probably pathogenic. We focus on the description of those variants that have been previously reported as Mendelian disease mutations that would increase the risk of mortality, such as degenerative diseases, neoplasm, and cardiac diseases. The observation of previously reported disease variants in centenarian genomes may aid clinical geneticists who are confronted with the challenge to evaluate their pathogenicity.

## Materials and Methods

### Study population

DNA samples of 44 AJ centenarians were collected as part of a longevity study at the Albert Einstein College of Medicine that was described elsewhere (Barzilai et al. 2003). The sample includes eight male (95–103 years old) and 36 female (95–106 years old) subjects. The mean and median age of the subjects was 99.6 and 100 years (standard deviation *σ* = 3.1). Informed written consent was obtained in accordance with the policy of the Committee on Clinical Investigations of the Albert Einstein College of Medicine. Genomic DNA extracted from blood cells using standard procedure was sent for sequencing. Mini-mental status examinations (MMSE) (Folstein et al. 1975) and selected health status information such as hearing, vision, and whether a subject had a history of cancer or myocardial infarction (MI) are available for the majority of the centenarians. The level of education was not available. Given that median MMSE scores are found to be a function of age and level of education (Crum et al. 1993; Bravo and Hebert 1997) and can be as low as 20 for people 85 years and older with <4 years of education (Crum et al. 1993), we use the reference score of ≥24 (one standard deviation of the mean MMSE score for people 85 and older; Bravo and Hebert 1997) to exclude the presence of dementia in our sample. To our knowledge, no reference MMSE scores specifically for centenarians are available.

### Genome sequencing and variant calling

Whole genome sequencing was performed on Illumina HiSeq 2000 platform (Illumina, San Diego, CA). Paired-end 2 × 100 reads were aligned to the GRCh37 human reference using the Burrows-Wheeler Aligner (BWA) (Li and Durbin 2009) and processed using the best-practices pipeline that includes marking of duplicate reads by the use of Picard tools (http://picard.sourceforge.net), and realignment around DNA insertions and deletions (indels) and base recalibration via Genome Analysis Toolkit (GATK) Lite ver. 2.3-9 (McKenna et al. 2010). Single-nucleotide variants (SNVs) were jointly called on the 44 unrelated AJ centenarians together with HapMap trio NA12877, NA12878, and NA12882 using the UnifiedGenotyper module of GATK Lite. The HapMap trio was sequenced at Illumina and released as part of the PlatinumGenomes project (http://www.illumina.com/platinumgenomes). We used variant quality score recalibration and Mendelian inconsistencies on the HapMap trio to determine the optimal variant quality score (VQSLOD) cutoff.

Joint calling for transcribed gene regions of 44 AJ centenarians identified a total of 6.3 million genetic variants. A total of 87,899 coding SNVs were found (100% call rate) in coding regions defined by the longest transcript according to the UCSC hg19 database (Meyer et al. 2012). For variants with a VQSLOD score >2, the concordance rate for SNVs between Illumina 2.5M chip and GATK variant calls was above 99.7% for all subjects. Due to the comparatively lower performance of the UnifiedGenotyper in calling short insertions and deletions, in the present study we decided not to analyze insertion/deletion variants.

### Database annotation of variants

Using custom scripts and the SNP & Variation Suite software (Version 7.7.8; Golden Helix, Inc., Bozeman, MT, http://www.goldenhelix.com), we annotated coding and splice variants based on the knownGene and the refGene tracks in the UCSC hg19 database (Meyer et al. 2012). To find variants of potential clinical relevance, variants were evaluated for their possible pathogenicity based on the August 2013 version of the ClinVar database (http://www.ncbi.nlm.nih.gov/clinvar/) (Riggs et al. 2013). This database provides free highly structured public access to clinically relevant sequence variants and it also provides evidence-based interpretation of clinical significance for each variant (Landrum et al. 2014). All ClinVar accession numbers for the identified variants are linked to the respective NCBI (The National Center for Biotechnology Information) database for reference sequences (RefSeq) as well as OMIM accession numbers. Population allele frequencies of variants were retrieved from the NHLBI Exome Sequencing Project (ESP) database (Exome Variant Server, NHLBI GO ESP, Seattle, WA) (http://evs.gs.washington.edu/EVS). To our knowledge, a similar large-scale population allele frequency data specific for the AJ population are currently not available.

### Quality control filtering of putative disease variants

A total of 225 autosomal and seven X-chromosomal coding SNVs were found to be annotated as “pathogenic” or “likely pathogenic” in the ClinVar database. Eighteen of these variants had a VQSLOD score <4, most often due to poor mapping quality indicating genomic segmental duplications. However, genomic regions that are difficult to map are also known to produce false-negative variant calls (Lee and Schatz 2012). Therefore, we performed manual evaluation of the 18 variants. These include two mutations in the gene *GBA* that are known to cause Gaucher disease as well as being associated with late-onset Parkinson's disease (most frequently referred to in the literature as L444P [uc001fjh.2:c.T1449C:p.L483P] and N370S [uc001fjh.2:c.A1227G:p.N409S]). Both mutations are known to be relatively frequent in the AJ population (Scott et al. 2010). Using BLAT (Kent 2002), N409S (rs76763715) was uniquely mapped to chromosome 1:155205634 and L483P (rs421016) was uniquely mapped to chr1:155205043. Thus, both variants are likely to be true SNVs in our sample and were therefore retained. The remaining 16 variants with low VQSLOD scores were removed.

## Results

In the coding regions of 44 centenarian genomes, we found 210 autosomal and six X-chromosomal SNVs that passed quality control. Among these, 207 variants were classified as pathogenic and nine as likely pathogenic based on the ClinVar database. Excluding the two *APOE* risk variants, 86 of these SNVs were found to be common defined as minor allele frequency (MAF) of 5% or higher in either the European or African American populations in the ESP database (Table S1). Due to their high allele frequency in the general population, these 86 variants are considered unlikely to be strongly pathogenic with high penetrance and therefore not further discussed. According to the OMIM database, of the remaining 130 variants (Tables 5 and S2), 39 were reported to cause autosomal dominant diseases, 72 to cause autosomal recessive diseases, 6 were associated with X-chromosomal inheritance, and 13 with other modes of inheritance such as digenic, imprinting, complex, or unclear mode of inheritance.

**Table 1:** Putative disease variants reported to cause degenerative diseases

CHR	POS	ID	REF	ALT	AC	Gene	Effect	CLNDBN	CLNACC	OMIM
1	155205043	rs421016	A	G	43/1/0	*GBA*	uc001fjh.2:c.T1449C:p.L483P	Gaucher's disease; late-onset Parkinson's disease; susceptibility to dementia with Lewy body	RCV000004511; RCV000004512	606463
1	155205634	rs76763715	T	C	43/1/0	*GBA*	uc001fjh.2:c.A1227G:p.N409S	Gaucher's disease; late-onset Parkinson's disease; susceptibility to dementia with Lewy body	RCV000004515; RCV000004516	606463
1	156146640	rs41265017	G	A	34/10/0	*SEMA4A*	uc001fnm.2:c.G2139A:p.R713Q	Retinitis pigmentosa 35	RCV000003528	607292
2	38298394	rs79204362	C	T	41/3/0	*CYP1B1*	uc002rqo.2:c.G1104Ap.R368H	Glaucoma early-onset digenic	RCV000008178	601771
5	110441839	rs35703638	G	A	43/1/0	*WDR36*	uc003kpd.2:c.G1346A:p.A449T	Glaucoma 1, open angle	RCV000001649	609669
5	110454719	rs34595252	A	G	43/1/0	*WDR36*	uc003kpd.2:c.A1974G:p.D658G	Glaucoma 1, open angle	RCV000001647	609669
8	55537560	rs77775126	C	T	43/1/0	*RP1*	uc003xsd.1 :c.C1119T:p.T373l	Retinitis pigmentosa 1	RCV000006334	603937
10	13178766	rs75654767	G	A	43/1/0	*OPTN*	uc001ilx1:c.G1635A:p.R545Q	Glaucoma 1, open angle	RCV000007515	602432
12	57431402	rs33962952	C	T	32/10/2	*MY01A*	uc001smw.3:c.G1986Ap.G662E	Deafness, autosomal dominant 48	RCV000008627	601478
12	57437119	rs55679042	C	T	43/1/0	*MY01A*	uc001 smw.3:c.G917A:p.V306M	Deafness, autosomal dominant 48	RCV000008625	601478
19	45411941	rs429358	T	C	39/4/1	*APOE*	uc002pab.2:c.T389C:p.C130R	Hyperlipoproteinemia, type 3, autosomal dominant; Alzheimer's disease associated with APOE4 variant	RCV000019438; RCV000019448	107741
19	45412079	rs7412	C	T	33/11/0	*APOE*	uc002pab.2:c.C527T:p.R176C	Familial type 3 hyperlipoproteinemia, associated with APOE2	RCV000019428	107741
20	25060096	rs74315433	C	T	43/1/0	*VSX1*	uc002wuf.2:c.G480Ap.G160D	Keratoconus 1	RCV000024251	605020
X	56591879	rs369947678	c	T	43/1/0	*UBQLN2*	uc004dus.2:c.C1574T:p.P525S	Amyotrophic lateral sclerosis 15 with or without frontotemporal dementia	RCV000022846	300264

CHR, chromosome; POS, position on chromosome based on GRCh37; ID, dbSNP ID; REF, reference sequence; ALT, alternative sequence; AC, counts of subjects with homozygous reference alleles/heterozygous/homozygous nonreference alleles; Effect, position of nucleotide change on UCSC transcript and amino acid change; CLNDBN and CLNACC, selected disease condition and accession ID from ClinVar; OMIM, OMIM accession numbers for the genes.

**Table 2:** Putative disease variants reported to cause neoplastic diseases

CHR	POS	ID	REF	ALT	AC	Gene	Effect	CLNDBN	CLNACC	OMIM
1	17354297	rs33927012	A	G	43/1/0	*SDHB*	uc001 bae.2:c.T488C:p.S163P	Cowden-like syndrome	RCV000013633	185470
1	182555149	rs74315364	C	A	43/1/0	*RNASEL*	uc001gpj.1:c.G794T:p.E265*	Prostate cancer, hereditary	RCV000013878	180435
5	112175240	rs1801166	G	C	43/1/0	*APC*	uc003kpy.3:c.G3950C:p.E1317Q	Adenomatous polyposis coli	RCV000000872	611731
8	16012594	rs41341748	G	A	43/1/0	*MSR1*	uc003wwz.2:c.C878T:p.R293*	Malignant tumor of prostate	RCV000015431	153622
10	43613908	rs77724903	A	T	42/2/0	*RET*	uc001jal.2:c.A2373T:p.Y791 F	Familial medullary thyroid carcinoma	RCV000014962	164761
11	67258382	rs104894190	G	A	41/3/0	*MP*	uc001olv.2:c.G912A:p.R304Q	Pituitary-dependent hypercortisolism	RCV000005171	605555
17	12899902	rs5030739	C	T	38/6/0	*ELAC2*	uc002gnz.3:c.G1622A:p.A541 T	Prostate cancer, hereditary	RCV000005359	605367
17	41226488	rs1800744	C	A	42/2/0	*BRCA1*	uc002ict.2:c.G4599T:p.S1533I	Familial cancer of breast	RCV000048588	113705
19	1223125	rs59912467	C	G	43/1/0	*STK11*	uc002lrl.1:c.C1063G:p.F355L	Peutz–Jeghers syndrome	RCV000007887	602216

CHR, chromosome; POS, position on chromosome based on GRCh37; ID, dbSNP ID; REF, reference sequence; ALT, alternative sequence; AC, counts of subjects with homozygous reference alleles/heterozygous/homozygous nonreference alleles; Effect, position of nucleotide change on UCSC transcript and amino acid change; CLNDBN and CLNACC, selected disease condition and accession ID from ClinVar; OMIM, OMIM accession numbers for the genes.

**Table 3:** Putative disease variants reported to cause autosomal dominant cardiac diseases

CHR	POS	ID	REF	ALT	AC	Gene	Effect	CLNDBN	CLNACC	OMIM
1	236918491	rs193922635	C	T	43/1/0	*ACTN2*	uc001hyf.2:c.C2148T:p.T716M	Cardiomyopathy	RCV000029298	102573
3	14180731	rs113449357	C	T	43/1/0	*TMEM43*	uc003byk.2:c.C935T:p.R312W	Cardiomyopathy	RCV000030555	612048
4	114294537	rs45454496	G	A	42/2/0	*ANK2*	uc003ibe.3:c.G11792A:p.E3931K	Cardiac arrhythmia, ankyrin B-related	RCV000019677	106410
12	2659186	rs121912775	G	A	43/1/0	*CACNA1C*	uc001qkl.2:c.G1469A:p.G490R	Brugada syndrome 3	RCV000019201	114205
12	22017410	rs61688134	C	T	42/2/0	*ABCC9*	uc001 rfh.2:c.G2201 A:p.V734l	Myocardial infarction 1	RCV000029274	601439
12	111356964	rs104894363	C	T	43/1/0	*MYL2*	uc001try.3:c.G38Ap.A13T	Familial hypertrophic cardiomyopathy 10	RCV000015108	160781
20	42744802	rs140740776	C	T	42/2/0	*JPH2*	uc002xli.1:c.G1514A:p.G505S	Familial hypertrophic cardiomyopathy 17	RCV000023411	605267
21	35742938	rs74315447	T	C	43/1/0	*KCNE2*	uc002ytt.1 :c.T162C:p.M54T	Long QT syndrome 6	RCV000006425	603796

CHR, chromosome; POS, position on chromosome based on GRCh37; ID, dbSNP ID; REF, reference sequence; ALT, alternative sequence; AC, counts of subjects with homozygous reference alleles/heterozygous/homozygous nonreference alleles; Effect, position of nucleotide change on UCSC transcript and amino acid change; CLNDBN and CLNACC, selected disease condition and accession ID from ClinVar; OMIM, OMIM accession numbers for the genes.

**Table 4:** Putative disease variants reported to cause other autosomal dominant diseases

CHR	POS	ID	REF	ALT	AC	Gene	Effect	CLNDBN	CLNACC	OMIM
1	152285861	rs61816761	G	A	42/2/0	*FLG*	uc001 ezu.1:c.C1502T:p.R501*	Ichthyosis vulgaris	RCV000017712	135940
2	167141109	rs41268673	G	T	36/7/1	*SCN9A*	uc010fpl.2:c.C1829A:p.P610T	Primary erythromelalgia	RCV000020511	603415
2	219755011	rs121908120	T	A	41/3/0	*WNT10A*	uc002yjd.1:c.T683Ap.F228l	Odontoonychodermal dysplasia	RCV000004717	606268
5	172662014	rs28936670	G	A	43/1/0	*NKX2-5*	uc003mcm.1:c.C74T:p.R25C	Tetralogy of Fallot	RCV000009572	600584
5	172662026	rs104893904	C	G	43/1/0	*NKX2-5*	uc003mcm.1:c.G62C:p.E21Q	Tetralogy of Fallot	RCV000009574	600584
6	32052313	rs121912575	C	T	42/2/0	*TNXB*	uc003nzl.2:c.G3323Ap.V1108M	Ehlers–Danlos syndrome type 3	RCV000009083	600985
8	11405576	rs55758736	G	A	42/2/0	*BLK*	uc003wty.2:c.G212Ap.A71T	Maturity-onset diabetes of the young type 11	RCV000013112	191305
8	18080001	rs4987076	G	A	39/5/0	*NAT1*	uc003wys.2:c.G632A:p.V211l	NAT1*7 ALLELE	RCV000019386	108345
8	55372085	NA	T	A	42/2/0	*SOX17*	uc003xsb.3:c.T776Ap.Y259N	Vesicoureteral reflux 3	RCV000001140	610928
8	106431420	rs121908601	A	G	42/2/0	*ZFPM2*	uc003ymd.2:c.A90G:p.E30G	Tetralogy of Fallot	RCV000006502	603693
12	6442643	rs4149584	C	T	41/3/0	*TNFRSF1A*	uc001qnu.2:c.G363A:p.R121Q	TNF receptor-associated periodic fever syndrome (TRAPS)	RCV000013134	191190
13	32351535	rs121918303	A	C	38/6/0	*RXFP2*	uc001 utt.2:c.A665C:p.T222P	Cryptorchidism, unilateral or bilateral	RCV000004376	606655
14	54418579	NA	T	C	43/1/0	*BMP4*	uc010aoh.2:c.A363G:p.H121R	Microphthalmia syndromic 6	RCV000022458	112262
15	74704267	rs56001514	G	A	43/1/0	*SEMA7A*	uc002axv.2:c.C1382T:p.R461C	Blood group John Milton Hagen system	RCV000029235	607961
16	1129586	rs121917877	C	T	42/2/0	*SSTR5*	uc002ckq.2:c.C719T:p.R240W	Resistance to somatostatin analog	RCV000013734	182455
17	72745313	rs35910969	C	G	43/1/0	*SLC9A3R1*	uc002jlo.2:c.C329G:p.L110V	Nephrolithiasis/osteoporosis, hypophosphatemic 2	RCV000005588	604990
18	58039060	rs121913563	C	T	43/1/0	*MC4R*	uc002lie.1:c.G524A:p.A175T	Obesity	RCV000015406	155541
19	11240278	rs137853964	G	A	43/1/0	*LDLR*	uc002mqk.3:c.G2480A:p.V827l	Familial hypercholesterolemia	RCV000030135	606945

CHR, chromosome; POS, position on chromosome based on GRCh37; ID, dbSNP ID; REF, reference sequence; ALT, alternative sequence; AC, counts of subjects with homozygous reference alleles/heterozygous/homozygous nonreference alleles; Effect, position of nucleotide change on UCSC transcript and amino acid change; CLNDBN and CLNACC, selected disease condition and accession ID from ClinVar; OMIM, OMIM accession numbers for the genes.

**Table 5:** Putative disease mutations reported to cause X-chromosomal diseases

CHR	POS	ID	REF	ALT	ALT_AC	ALT_AC (male)	Gene	Effect	CLNDBN	CLNACC	OMIM
X	31496398	rs1800279	T	C	4	1	*DMD*	uc004dda.1:c.A8763G:p.H2921R	Becker muscular dystrophy	RCV000012020	300377
X	31496426	rs1800278	T	C	1	1	*DMD*	uc004dda.1:c.A8735G:p.N2912D	Duchenne muscular dystrophy	RCV000012019	300377
X	31496431	rs41305353	T	A	1	1	*DMD*	uc004dda.1:c.A8730T:p.E2910V	Duchenne muscular dystrophy	RCV000012018	300377
X	84563194	rs75398746	C	T	2	0	*POF1B*	uc004eer.2:c.G987A:p.R329Q	Premature ovarian failure 2b	RCV000011541	300603
X	105278361	rs1804495	C	A	11	1	*SERPINA7*	uc004eme.1:c.G910T:p.L304F	Thyroxine-binding globulin variant p	RCV000010442	314200

CHR, chromosome based on GRCh37; POS, position on chromosome; ID, dbSNP ID; REF, reference sequence; ALT, alternative sequence; ALT_AC, nonreference allele counts; Effect, position of nucleotide change on UCSC transcript and amino acid change; CLNDBN and CLNACC, selected disease condition and accession ID from ClinVar; OMIM, OMIM accession numbers for the genes.

### Variants reported as causal for degenerative diseases of advanced age

Variants associated with age-related degenerative diseases were found in the genes *APOE*, *GBA*, *UBQLN2*, *SEMA4A*, *RP1*, *MYO1A*, *CYP1B1*, *OPTN*, *VSX1*, and *WDR36* (Table 1).

Given the important role of *APOE* variants in late-onset Alzheimer's disease, we first looked at the *APOE ε*4 allele as defined by the ancestral alleles rs429358-C and rs7412-C (130Arg and 176Arg) and *APOE ε*2 allele as defined by rs429358-T and rs7412-T (130Cys and 176Cys). In our centenarian sample, the allele frequencies for *APOE ε*4, *ε*3, and *ε*2 were 6.8%, 80.7%, and 12.5%, respectively. One of the two *ε*2/*ε*4 and one of the two *ε*3/*ε*4 heterozygous carriers were found to have advanced dementia of unknown etiology and the other two subjects were cognitively intact. Notably, the centenarian homozygous for the *ε*4 allele had an MMSE score of 25 at age 97. Neither this centenarian nor any other subject in our study carried the protective variant A673T in the *APP* gene (Jonsson et al. 2012). We also observed one carrier for each of the known *GBA* mutations L483P (also known as L444P) (rs421016) and N409S (N370S) (rs76763715), which is in line with the reported frequency of 3% for either mutation in AJ controls (Sidransky et al. 2009).

On the X chromosome we identified a female centenarian with a previously described mutation in *UBQLN2* P525S (Deng et al. 2011), which was found to cause an X-linked dominant type of familial amyotrophic lateral sclerosis (ALS) and ALS/dementia with an estimated penetrance of approximately 90% (Table 1). This subject had normal MMSE (score = 28) at age 102 without any neurological symptoms.

Furthermore, disease alleles were observed for other autosomal dominantly inherited degenerative diseases of sensory function, including retinitis pigmentosa (*SEMA4A*, *RP1*), deafness (*MYO1A*), glaucoma (*CYP1B1*, *OPTN*, *WDR36*), and keratoconus (*VSX1*). Of note, none of the 10 centenarians carrying the *SEMA4A* R713Q had vision impairment but the single subject carrying the *RP1* T373I was blind. No hearing impairment was noticed in one of two subjects homozygous and four of ten heterozygous carriers of the *MYO1A* G662E variant that was linked to an autosomal dominant form of deafness.

### Variants for neoplastic diseases

We found variants in the five genes *APC*, *BRCA1*, *RET*, *RNASEL*, and *STK11* that were linked to autosomal dominant forms of cancer or neoplasm as well as four complex risk variants in *ELAC2*, *MSR1*, *AIP*, and *SDHB* (Table 2). The clinical relevance of these variants has been discussed in the literature and their presence in the genomes of centenarians indicates that these variants are compatible with exceptional longevity.

The tumor suppressor gene *STK11* variant F355L (rs59912467) linked to Peutz–Jeghers syndrome was seen in one centenarian. It was reported to affect both the AMPK pathway and cell polarity, thus contributing to the development of malignancies (Forcet et al. 2005). Notably, the same subject carried two additional variants linked to thyroid carcinoma (*RET* Y791F, rs77724903) and Cowden-like syndrome (*SDHB* S163P, rs33927012), but was free of any type of neoplasia at age 97. The Y791F variant in the *RET* protooncogene was further present in another centenarian with a history of cancer of unknown origin. This variant was first described in two German families with familial medullary thyroid carcinoma (Berndt et al. 1998). Six centenarians carried the A541T variant in the gene *ELAC2* (rs5030739) that was reported to be associated with prostate cancer (Rebbeck et al. 2000; Tavtigian et al. 2001; Camp and Tavtigian 2002). One of the two male carriers for A541T had no cancer at age 103 years and the other had an unknown type of cancer.

### Variants for autosomal dominant forms of cardiac disease

We found variants in *ABCC9*, *ACTN2*, *ANK2*, *CACNA1C*, *JPH2*, *KCNE2*, *MYL2*, and *TMEM43* that were linked to autosomal dominant phenotypes affecting cardiac function (Table 3).

Three of the variants in these genes are annotated as causes of cardiac arrhythmia with increased risk of sudden cardiac death. Two centenarians were heterozygous for the E3931K variant in *ANK2* (rs45454496) variant, also known as E1813K, which was reported as a loss-of-function mutation in the ankyrin-B regulatory domain (Mohler et al. 2003, 2004). One centenarian carried the G490R variant in *CACNA1C* (rs121912775), a loss-of-function change that was linked to Brugada syndrome 3 (Antzelevitch et al. 2007). Another carried the M54T (rs74315447) in *KCNE2*, which was reported to alter the transmembrane domain of MiRP1 that reduces potassium currents leading to long QT syndrome and ventricular fibrillation (Abbott et al. 1999; Splawski et al. 2000).

Five variants linked to cardiomyopathy were found in our centenarian sample. The *MYL2* A13T (rs104894363) variant located in the regulatory light chain of myosin was reported to be causal for a subtype of familial hypertrophic cardiomyopathy with onset of clinical symptoms around middle age (Poetter et al. 1996; Andersen et al. 2001). The gene *ABCC9* encodes the regulatory *SUR2A* subunit of the cardiac ATP-sensitive potassium (K_ATP_) channel. Two centenarians carried the *ABCC9* V734I variant (rs61688134), which was reported to increase the risk of MI by 6.40-fold. (Minoretti et al. 2006). The G505S (rs140740776) variant in *JPH2* was reported to be associated with hypertrophic cardiomyopathy in a relatively small sample of Japanese patients (Matsushita et al. 2007). One centenarian carried the *TMEM43* R312W variant linked to autosomal dominant arrhythmogenic right ventricular cardiomyopathy/dysplasia (Haywood et al. 2013). Interestingly, one centenarian was found to be heterozygous for both *JPH2* G505S and *ABCC9* V734I and another was heterozygous for both *JPH2* G505S and *ANK2* E3931K.

### Variants for other autosomal dominant and X-chromosomal diseases

We found 18 rare ClinVar variants for other autosomal dominant diseases (Table 4) and six mutations for X-chromosomal diseases (Table 5). These include variants that increase the risk of metabolic disorders, including hypercholesterolemia (*LDLR*, V827I, rs137853964), maturity-onset diabetes of the young (MODY) (*BLK*, A71T, rs55758736), and obesity (*MC4R*, A175T, rs121913563). One male and one female centenarian carried the V1108M variant in *TNXB* (rs121912575) linked to the dominant form of Ehlers–Danlos syndrome type 3 (Zweers et al. 2005). The remaining autosomal mutations are annotated to cause a wide range of dominantly inherited phenotypes including metabolic, genitourinary, and skin conditions as well as pediatric conditions, including developmental syndromes, for example, the tetralogy of Fallot, a severe malformation that would reduce life span. Three centenarians presented a variant in *TNFRSF1A* (R121Q, rs4149584) in the heterozygous state that was linked to TNF-receptor associated periodic fever. This was the only variant for dominantly inherited immune disease in this dataset.

Aside from the above mentioned mutation in *UBQLN2*, the observed X-chromosomal variants include three variants in the *DMD* gene that have been discussed as cause of Duchenne and Becker muscular dystrophy. One frequent missense variant rs1800279 (H2921R) in the *DMD* gene was observed in one male centenarian and another male centenarian carried both rare variants rs1800278 (N2912D) and rs41305353 (E2910V). The carriers did not have any documented muscle diseases.

### Variants for recessive diseases and complex diseases

Among the 72 variants for recessive traits, we found three variants that were observed in the homozygous state in at least one centenarian (Table S2). These are variants in *ADA*, *ALG6*, and *HPS5*. The *ALG6* variant Y131H (rs35383149) has been annotated to cause congenital disorder of glycosylation, type Ic, a childhood onset metabolic disorder accompanied by severe neurological symptoms (Miller et al. 2011). We found no literature evidence supporting the pathogenicity of the *ADA* variant D8N (rs73598374) or the *HPS5* variant T1098I (rs61884288).

Three subjects were heterozygous carriers for Factor V Leiden (*F5*, Q534R, rs6025) (Bertina et al. 1994). Heterozygous carriers have elevated risks of deep venous thrombosis, pulmonary embolism (Juul et al. 2004), and stroke (Casas et al. 2004). Note that the GRCh37 genome assembly presents the risk allele as the reference allele and no individuals homozygous for the reference allele were seen in our sample. One carrier had a history of MI and another had a history of both MI and stroke, but the third carrier had no known thrombotic diseases by the age of 106 years.

Finally, we also identified homozygous individuals for putative recessive disease alleles with relevance for longevity regardless of their population allele frequency. Of note, we found three missense variants in the *ACADS* gene R107C (rs61732144), R171W (rs1800556), and G209S (rs1799958) (Tables S1 and S2) that were initially reported (Corydon et al. 2001; Pedersen et al. 2008) in 10 patients with ethylmalonic aciduria and short-chain acyl-CoA dehydrogenase (SCAD) deficiency, a mitochondrial fatty acid oxidation disorder causing neuromuscular phenotypes with hypotonia and developmental delay as the prominent features of the disease. Homozygosity for R107C as well as compound heterozygosity for R107C and G209S have been described as disease causing in AJ patients with SCAD with reduced penetrance (Tein et al. 2008) depending on other genetic modifiers or environmental stressors. Variants R171W and G209S have both high allele frequencies (5.5% and 23.5%, respectively) in the general population (U.S. and the Netherlands) and they are considered to confer susceptibility for clinical disease (van Maldegem et al. 2006). We found five AJ centenarians who are homozygous for the variant G209S (MAF = 36.4%) and they do not carry any other missense variants in the *ACADS* gene. The carrier frequency for the R107C variant in our centenarians (3/44) is identical to the reported carrier frequency in AJ population (Tein et al. 2008).

## Discussion

In this study we observed many previously reported Mendelian mutations that are sufficiently benign to allow the individual carriers to achieve exceptional longevity. The presence of these specific variants in the genomes of centenarians can be helpful for clinical geneticists who are challenged with the evaluation of their putative pathogenicity as incidental findings. More generally, our findings support the notion that for many putative disease variants it is not straightforward to decide whether they should be regarded and acted upon as incidental findings, when they are observed in healthy individuals (Kingsmore 2013). Several genes harboring putatively pathogenic variants in our centenarians are on the list of 57 genes with reportable findings according to the ACMG recommendations (Green et al. 2013b). These genes comprise 4 of the 24 genes for cancer (*BRCA1*, *APC*, *RET*, *STK11*), 1 of the 20 cardiac disease genes (*TMEM43*), 1 of the remaining 13 genes (*LDLR*). This demonstrates the challenge to identify actionable mutations even in well-established disease genes. The variants found in four of the cancer genes *APC*, *BRCA1*, *RET*, and *SDHB* in our AJ centenarian sample had also been labeled as “almost certainly benign” in another sequencing study (ClinSeq) of 572 middle aged participants (17% of which are of AJ ancestry) (Johnston et al. 2012). Surprisingly, we also observed many rare variants linked to dominantly inherited forms of diseases that would clearly affect life expectancy. These disease traits include cardiac diseases such as cardiomyopathy and arrhythmia that increase the risk of sudden cardiac death as well as metabolic diseases such as diabetes, hypercholesterolemia, and obesity. One explanation for the presence of disease alleles in centenarians might be that these variants have incomplete penetrance as a result of complex interplay between modifying genetic and environmental factors (Bergman et al. 2007; Cooper et al. 2013). However, for some variants the published evidence may be viewed as too weak to uphold their classification as pathogenic. For example, the three missense variants in the *DMD* gene were all found in at least one male centenarian. Given the markedly reduced life expectancy of patients affected by Duchenne muscular dystrophy (Kieny et al. 2013), it is unlikely that these variants are pathogenic. In other cases, the resulting phenotypes of mutations could have little impact on longevity, which may primarily apply to diseases such as deafness, glaucoma, retinitis pigmentosa, premature ovarian failure, or ichthyosis. In other instances the possible influence on life expectancy is not clear, such as the variant V1108M in the *TNXB* gene, which was reported to cause Ehlers–Danlos syndrome type 3 (Zweers et al. 2005) and shown to affect the protein function (Zhuang et al. 2010).

We note that many presumable disease mutations are present in centenarians with fairly high frequency. We did not discuss these variants in detail because they are common in the ESP data. With the exception of the APOE *ε*4 allele, these common variants are from the beginning more likely to constitute polymorphisms than any clinically significant mutations. Despite its high population frequency, the APOE *ε*4 allele was suggested to follow semi-dominant inheritance (Genin et al. 2011). *APOE* is also the only reported gene reaching GWAS significance threshold for longevity (Beekman et al. 2013). In our centenarian sample, the APOE *ε*4 allele frequency is very similar to the previously reported allele frequencies in 325 French Caucasian centenarians (Schachter et al. 1994), namely 5.2% for APOE *ε*4 and 12.8% for APOE *ε*2. In the ESP dataset, the respective allele frequencies for *ε*4 and *ε*2 are 11.7% and 5.6% in European Americans and 18.9% and 8.7% in African Americans. This is congruent with the previous finding that *ε*4 was associated with reduced and *ε*2 with increased life span (Deelen et al. 2011). However, earlier studies did not observe any homozygote centenarians for APOE *ε*4. As would be expected, the nonaffected APOE *ε*4 homozygote had rare coding variants in APOE modifier candidate genes, but testing their significance needs to be left to subsequent studies. The future identification of such protective genetic factors in centenarians may increase the clinical utility of the APOE *ε*4 allele. Due to the relatively small sample size and lack of a control data set from a matched population, we cannot search for novel variants with impact on longevity here.

In future, personalized genomic sequencing is likely to generate many incidental findings. Rigorous investigations for cosegregation in family members, large ancestry-matched population screening, and functional studies will be required to identify those variants that are sufficiently penetrant to be considered clinically relevant. Genetic counseling provided by trained clinicians will be necessary to communicate the potential impact of genetic variants to avoid unnecessary and far reaching burden. This is especially true in considering genetic screening in the young, where the demarcation of putatively actionable mutations needs to be approached with considerable caution, allowing appropriate balance to be conveyed in risk/benefit discussions with parents. Our study provides a list of variants that are currently flagged as pathogenic, but are compatible with exceptional longevity. Thereby, our study contributes to the genetic community's efforts to refine the set of variants with strong clinical significance and mark previously reported disease mutations with unclear clinical significance.

## Supporting Information

**Table S1.** Putative disease alleles found in AJ Centenarians that have minor allele frequency ≥5% in either Europeans or African Americans from the Exome Sequencing Project (ESP).

**Table S2.** Putative disease variants reported to cause autosomal recessive diseases and diseases with other mode of inheritance.
