# Supplementary material for: Disease variants in genomes of 44 centenarians
Source: Mol Genet Genomic Med. 2014 Jun 15;2(5):438–50. doi: 10.1002/mgg3.86 (PMC4190879; doi:10.1002/mgg3.86)
Supplement: Supplementary file 1 — Table S1. Putative disease alleles found in AJ Centenarians that have minor allele frequency ≥5% in either Europeans or African Americans from the Exome Sequencing Project (ESP). [file mgg30002-0438-SD1.pdf]

Supplementary Table 1

CHR	POS	ID	CLNDBN	CLNACC	ESP6500_ AA / %	ESP6500_ EA / %
1	11856378	rs1801133	MTHFR deficiency thermolabile type	RCV000003697.2	12.2	34.7
1	31349647	rs2491132	Obesity association with	RCV000013593.1	4.2	21.1
1	70904800	rs1021737	Homocysteine total plasma elevated	RCV000003075.1	10.8	30.4
1	98348885	rs1801265	Dihydropyrimidine dehydrogenase deficiency	RCV000000464.1	60.0	77.5
1	100672060	rs12021720	Intermediate maple syrup urine disease type 2	RCV000012727.1	77.5	90.2
1	115236057	rs17602729	Muscle AMP deaminase deficiency	RCV000019933.1	2.4	13.1
1	156848918	rs6336	Familial medullary thyroid carcinoma	RCV000013100.1	1.2	6.0
1	156848946	rs6339	Familial medullary thyroid carcinoma	RCV000013101.1	1.2	6.0
1	171080080	rs1736557	Trimethylaminuria	RCV000017698.1	3.7	6.9
1	203194186	rs2297950	Chitotriosidase deficiency	RCV000010134.1	27.0	29.3
1	231408091	rs11558492	Rhizomelic chondrodysplasia punctata type 2	RCV000029140.1	9.2	20.6
2	44066247	rs11887534	Gallbladder disease 4	RCV000005263.1	5.2	5.5
2	136590746	rs3754689	Congenital lactase deficiency	RCV000049806.1	44.4	18.1
2	202074098	rs13010627	Autoimmune lymphoproliferative syndrome type 2	RCV000020930.1	1.6	6.6
3	8775661	rs1008642	Distal myopathy Tateyama type	RCV000008786.1	0.0	23.9
3	10331457	rs696217	Obesity	RCV000005365.1	2.3	7.7
3	38645420	rs1805124	Progressive familial heart block type 1A	RCV000010000.1	27.5	23.2
3	45814094	rs17279437	Hyperglycinuria	RCV000005117.1	2.0	9.9
3	46399208	rs1799864	Congenital human immunodeficiency virus	RCV000008756.1	15.8	9.2
3	122003757	rs1801725	Serum calcium level	RCV000008854.1	4.1	14.8
3	133494354	rs1049296	Transferrin variant c1/c2	RCV000013451.1	7.7	15.8
3	165491280	rs1803274	Bche k variant	RCV000014120.1	19.0	20.2
4	88533540	rs36094464	Dentinogenesis imperfecta - Shield's type II	RCV000018354.1	30.9	6.5
4	102751076	rs10516487	Systemic lupus erythmatosus association with	RCV000001331.1	24.4	30.3
4	187113041	rs1055138	Bietti crystalline corneoretinal dystrophy	RCV000032544.2	41.3	48.9
4	187158034	rs3733402	Prekallikrein deficiency	RCV000012817.1	73.6	50.7
5	33951693	rs16891982	Skin/hair/eye pigmentation variation in 5	RCV000004763.1	18.5	95.9
5	35861068	rs1494558	Severe combined immunodeficiency autosomal recessive T cell-negative B cell-positive NK cell-positive	RCV000015964.1	75.4	67.4

5	35871190	rs1494555	Severe combined immunodeficiency autosomal recessive T cell-negative B cell-positive NK cell-positive	RCV000015965.1	87.4	68.0
5	118811533	rs25640	Bifunctional peroxisomal enzyme deficiency	RCV000008096.1	17.6	46.0
5	149212243	rs7732671	Obesity variation in	RCV000002119.1	16.1	8.4
5	176520243	rs351855	Cancer progression and tumor cell motility	RCV000017723.1	12.9	30.1
6	18130918	rs1142345	Thiopurine methyltransferase deficiency	RCV000013559.1	5.3	4.2
6	26093141	rs1800562	Hereditary hemochromatosis|Porphyria cutanea tarda susceptibility to|Porphyria variegata susceptibility to|Hemochromatosis juvenile digenic|Alzheimer disease susceptibility to|Transferrin serum level quantitative trait locus 2|Microvascular complications of diabetes 7	RCV000000019.2	1.5	6.4
6	49580247	rs16879498	Rh-null hemolytic anemia regulator type	RCV000013940.1	6.7	2.7
7	100771717	rs6092	Plasminogen activator inhibitor type 1 deficiency	RCV000014541.1	2.3	11.7
7	138417791	rs3807153	Renal tubular acidosis distal autosomal recessive	RCV000005461.1	18.5	4.8
7	141672604	rs10246939	Phenylthiocarbamide tasting	RCV000003040.1	47.8	45.6
8	21976710	rs7014851	Alopecia universalis congenita	RCV000007755.1	25.0	1.6
8	27373865	rs751141	Hypercholesterolemia familial due to ldlr defect modifier of	RCV000018074.1	9.4	10.7
9	120475302	rs4986790	Endotoxin hyporesponsiveness|MACULAR DEGENERATION AGE-RELATED 10 SUSCEPTIBILITY TO|Colorectal cancer susceptibility to	RCV000007040.1	7.1	6.0
9	120475602	rs4986791	Endotoxin hyporesponsiveness	RCV000007043.1	1.7	6.3
9	132580901	rs1801968	Dystonia 1 torsion modifier of	RCV000005490.1	2.7	14.0
9	136301982	rs2301612	Upshaw-Schulman syndrome	RCV000006169.1	9.6	40.6
10	54531226	rs1800451	Mannose-binding protein deficiency	RCV000015425.1	22.7	1.8
10	54531235	rs1800450	Mannose-binding protein deficiency	RCV000015424.1	2.9	14.0
10	54531242	rs5030737	Mannose-binding protein deficiency	RCV000015426.1	1.2	6.9
10	70645376	rs10509305	Preeclampsia/eclampsia 4	RCV000001790.1	5.8	21.5
10	115804036	rs1801252	Resting heart rate	RCV000019323.1	19.4	10.8
11	13514053	rs6256	Primary hyperparathyroidism	RCV000014767.1	0.0	15.4
11	68562328	rs2229738	Carnitine palmitoyltransferase I deficiency	RCV000055868.1	1.4	7.7
11	68846399	rs35264875	Skin/hair/eye pigmentation variation in 10	RCV000000763.1	3.5	17.7
11	68855363	rs3829241	Skin/hair/eye pigmentation variation in 10	RCV000000764.1	10.6	38.3
11	88911696	rs1042602	Skin/hair/eye pigmentation variation in 3	RCV000003977.2	7.6	37.7
11	89017961	rs1126809	Oculocutaneous albinism type 1B	RCV000003978.2	5.1	28.1

11	113270828	rs1800497	Dopamine receptor d2 reduced brain density of	RCV000002186.1	32.7	19.0
12	10271087	rs16910526	Candidiasis familial 4	RCV000004721.1	2.6	7.9
12	14993439	rs11276	DOMBROCK BLOOD GROUP	RCV000019304.1	27.9	39.5
12	121176083	rs1799958	Deficiency of butyryl-CoA dehydrogenase	RCV000004035.1	6.4	26.5
12	122295335	rs1154510	4-Alpha-hydroxyphenylpyruvate hydroxylase deficiency	RCV000001643.1	95.6	87.3
13	108863591	rs1805388	Multiple myeloma resistance to	RCV000008116.1	10.3	15.5
13	108863609	rs1805389	Multiple myeloma resistance to	RCV000008115.1	1.2	5.1
14	21790040	rs10151259	Cone-rod dystrophy 13	RCV000005275.1	20.5	24.5
14	21811196	rs17103671	Leber congenital amaurosis 6	RCV000005276.1	7.4	0.4
15	28230318	rs1800407	Skin/hair/eye pigmentation variation in 1	RCV000001014.1	1.2	7.8
15	28260053	rs1800401	Skin/hair/eye pigmentation variation in 1	RCV000001013.1	13.4	5.0
16	16251599	rs2238472	Pseudoxanthoma elasticum	RCV000006948.1	10.8	28.3
16	27374180	rs1805015	Atopy resistance to	RCV000015778.1	36.3	16.2
16	48258198	rs17822931	Apocrine gland secretion variation in	RCV000003737.1	3.1	13.2
16	53720436	rs61747071	Retinitis pigmentosa in ciliopathies modifier of	RCV000001133.1	10.1	3.3
17	3397702	rs12948217	Spongy degeneration of central nervous system	RCV000002727.1	0.0	31.2
17	12915009	rs4792311	Prostate cancer hereditary 2	RCV000005358.1	23.5	29.7
17	41244435	rs16941	Familial cancer of breast	RCV000048070.1	18.8	32.5
17	42338945	rs5036	Band 3 memphis	RCV000019328.1	10.2	2.3
17	42453065	rs5911	Bak platelet-specific antigen	RCV000003025.1	39.0	37.4
17	48437456	rs6504649	Pseudoxanthoma elasticum modifier of severity of	RCV000002642.1	24.1	39.8
18	29104714	rs2230234	Cardiomyopathy	RCV000029672.1	1.4	8.6
19	5844792	rs28362459	Le(-) PHENOTYPE	RCV000019289.1	28.2	9.8
19	41858921	rs1800470	Cystic fibrosis	RCV000013360.1	0.0	55(dbSNP)
19	45316804	rs3810141	BLOOD GROUP--LUTHERAN NULL	RCV000000471.1	0.0	6.1
20	3193842	rs1127354	Inosine triphosphatase deficiency	RCV000015867.1	4.2	6.8
20	23618427	rs1064039	Age-related macular degeneration 11	RCV000005989.1	18.4	17.5
21	46931109	rs12483377	Knobloch syndrome 1	RCV000018655.1	3.6	8.8
22	18901004	rs450046	Proline dehydrogenase deficiency	RCV000004222.1	83.6	93.9
22	46731689	rs11090865	Deafness mitochondrial modifier of	RCV000001353.1	20.3	9.9
22	51064039	rs743616	Metachromatic leukodystrophy	RCV000020311.1	39.4	53.0
