# Supplementary material for: Disease variants in genomes of 44 centenarians
Source: Mol Genet Genomic Med. 2014 Jun 15;2(5):438–50. doi: 10.1002/mgg3.86 (PMC4190879; doi:10.1002/mgg3.86)
Supplement: Supplementary file 2 — Table S2. Putative disease variants reported to cause autosomal recessive diseases and diseases with other mode of inheritance. [file mgg30002-0438-SD2.pdf]

Supplementary Table 2

CHR	POS	ID	REF	ALT	AC	GENE	EFFECT	CLNDBN	CLNACC
1	31347320	rs2282440	G	A	42/2/0	*SDC3*	uc001bse.2:c.C987T:p.T329I	Obesity association with	RCV000013592
1	46655645	rs74374973	C	T	39/5/0	*POMGNT1*	uc001cpe.2:c.G1667A:p.D556N	Limb-girdle muscular dystrophy-dystroglycanopathy type C3	RCV000004204
1	53668099	rs74315294	C	T	41/3/0	*CPT2*	uc001cvb.3:c.C339T:p.S113L	Carnitine palmitoyltransferase II deficiency late-onset	RCV000009510
1	63872032	rs35383149	T	C	35/8/1	*ALG6*	uc010oow.1:c.T392C:p.Y131H	Congenital disorder of glycosylation type 1C	RCV000023375
1	68910315	rs61752878	C	T	40/4/0	*RPE65*	uc001dei.1:c.G395A:p.A132T	Retinitis pigmentosa 20	RCV000013998
1	70881670	rs28941785	C	T	43/1/0	*CTH*	uc001dfd.2:c.C201T:p.T67I	Cystathioninuria	RCV000003073
1	94517254	rs76157638	C	G	43/1/0	*ABCA4*	uc001dqh.2:c.G2589C:p.G863A	Stargardt disease 1	RCV000008328
1	156146640	rs41265017	G	A	34/10/0	*SEMA4A*	uc001fnm.2:c.G2139A:p.R713Q	Retinitis pigmentosa 35	RCV000003528
1	158624528	rs35948326	G	T	40/4/0	*SPTA1*	uc001fst.1:c.C2910A:p.A970D	Spherocytosis type 3 autosomal recessive	RCV000013699
1	159175494	rs34599082	C	T	42/2/0	*DARC*	uc001fto.2:c.C266T:p.R89C	Duffy blood group system fy(bwk) phenotype	RCV000000009
1	169519049	rs6025	T	C	0/3/41	*F5*	uc001ggg.1:c.A1602G:p.Q534R	Thrombophilia due to factor V leiden	RCV000000674
3	14200382	rs74737358	G	T	43/1/0	*XPC*	uc011ave.1:c.C1002A:p.P334H	Xeroderma pigmentosum group C	RCV000000277
3	15686693	rs13078881	G	C	40/4/0	*BTD*	uc003cah.2:c.G1331C:p.D444H	Biotinidase deficiency	RCV000001977
3	52023042	rs121912701	G	A	43/1/0	*ACY1*	uc003dcp.2:c.G1179A:p.R393H	Aminoacylase 1 deficiency	RCV000019742
3	128622922	rs115532916	G	A	42/2/0	*ACAD9*	uc003ela.3:c.G977A:p.A326T	Acyl-CoA dehydrogenase family member 9 deficiency of	RCV000023868
3	150690352	rs28939091	A	C	42/2/0	*CLRN1*	uc003eyk.1:c.T145G:p.N49K	Usher syndrome type 3	RCV000004645
3	165548529	rs1799807	T	C	41/3/0	*BCHE*	uc003fem.3:c.A294G:p.D98G	Apnea postanesthetic due to bche atypical-1	RCV000014102
4	187195347	rs121965063	G	T	43/1/0	*F11*	uc003iza.1:c.G404T:p.E135*	Hereditary factor XI deficiency disease	RCV000012666
4	187201412	rs121965064	T	C	41/3/0	*F11*	uc003iza.1:c.T902C:p.F301L	Hereditary factor XI deficiency disease	RCV000012667
5	1294166	rs61748181	C	T	42/2/0	*TERT*	uc003jcb.1:c.G836A:p.A279T	Aplastic anemia	RCV000032399
5	33963870	rs26722	C	T	42/2/0	*SLC45A2*	uc003jid.2:c.G815A:p.E272K	Skin/hair/eye pigmentation variation in 5	RCV000004762
5	60240799	rs121434324	C	A	42/2/0	*ERCC8*	uc003jsm.3:c.G38T:p.E13*	Cockayne syndrome type A	RCV000001786
5	73981270	rs820878	T	C	0/4/40	*HEXB*	uc003kdf.3:c.T186C:p.L62S	Sandhoff disease infantile type,.	RCV000004086
5	150723155	rs77010315	C	A	43/1/0	*SLC36A2*	uc003lty.2:c.G261T:p.G87V	Iminoglycinuria digenic	RCV000002483
5	151202476	rs116474260	C	T	42/2/0	*GLRA1*	uc003lut.2:c.G1133A:p.G378S	Hyperekplexia hereditary	RCV000031885

6	18139228	rs1800460	C	T	43/1/0	*TPMT*	uc003ncm.2:c.G461A:p.A154T	Thiopurine methyltransferase deficiency	RCV000013559
6	29641105	rs118204431	G	A	42/2/0	*ZFP57*	NP_001103279.2:p.Cys261Ter	Transient_neonatal_diabetes_mellitus_1	RCV000000751
6	31827947	rs104893981	G	A	43/1/0	*NEU1*	uc003nxq.3:c.C894T:p.A298V	Sialidosis type II	RCV000002561
7	87082273	rs58238559	T	C	43/1/0	*ABCB4*	uc003uiw.1:c.A524G:p.T175A	Cholecystitis	RCV000014690
7	107555951	rs121964990	G	T	43/1/0	*DLD*	uc003vet.2:c.G686T:p.G229C	Maple syrup urine disease type 3	RCV000012744
7	117282620	rs77010898	G	A	43/1/0	*CFTR*	uc003vjd.2:c.G3847A:p.W1283*	Cystic fibrosis	RCV000007549
8	19813529	rs268	A	G	43/1/0	*LPL*	uc003wzk.3:c.A954G:p.N318S	Hyperlipidemia familial combined	RCV000001615
8	77896060	rs61752123	G	A	43/1/0	*PXMP3*	uc003yay.2:c.C356T:p.R119*	Peroxisome biogenesis disorder 5A	RCV000014703
9	104189856	rs1800546	C	G	43/1/0	*ALDOB*	uc004bbk.2:c.G449C:p.A150P	Hereditary fructosuria	RCV000000493
9	133333936	rs35269064	G	T	43/1/0	*ASS1*	uc004bzm.2:c.G324T:p.R108L	Citrullinemia type I	RCV000006706
9	136302063	rs11575933	C	T	43/1/0	*ADAMTS13*	uc004cdv.3:c.C1424T:p.P475S	Upshaw-Schulman syndrome	RCV000006170
10	14977469	rs41297018	C	T	43/1/0	*DCLRE1C*	uc001inn.2:c.G458A:p.G153R	Severe combined immunodeficiency disease	RCV000029656
10	72643763	rs104894172	C	A	43/1/0	*PCBD1*	uc001jrn.1:c.G260T:p.E87*	Hyperphenylalaninemia bh4-deficient d	RCV000018286
10	96522463	rs28399504	A	G	42/2/0	*CYP2C19*	uc010qnz.1:c.A2G:p.M1V	Mephenytoin poor metabolism of	RCV000018399
10	101829514	rs61751507	C	T	39/5/0	*CPN1*	uc001kql.2:c.G534A:p.G178D	Anaphylotoxin inactivator deficiency	RCV000007002
10	115348046	rs7080536	G	A	43/1/0	*HABP2*	uc001lai.3:c.G1602A:p.G534E	Factor VII Marburg I Variant Thrombophilia	RCV000006338
11	5255582	rs35152987	C	A	43/1/0	*HBD*	uc001maf.1:c.G83T:p.A28S	delta Thalassemia|HEMOGLOBIN A(2) YIALOUSA	RCV000016221
11	18303533	rs61884288	G	A	40/3/1	*HPS5*	uc001mod.1:c.C3294T:p.T1098I	Hermansky-Pudlak syndrome 5	RCV000021032
11	36615502	rs193922574	G	A	43/1/0	*RAG2*	uc001mwv.3:c.C218T:p.R73C	Severe combined immunodeficiency disease	RCV000030398
11	89017973	rs62645921	C	T	43/1/0	*TYR*	uc001pcs.2:c.C1218T:p.P406L	Oculocutaneous albinism type 1B	RCV000003976
11	134132450	rs121908419	G	A	43/1/0	*ACAD8*	uc001qhk.2:c.G1130A:p.G377S	Deficiency of isobutyryl-CoA dehydrogenase	RCV000005687
12	52760957	rs61630004	C	T	34/10/0	*KRT85*	uc001sag.2:c.G234A:p.R78H	Ectodermal dysplasia 'pure' hair-nail type	RCV000007238
12	53343340	rs57758506	A	T	39/5/0	*KRT18*	uc001sbg.2:c.A384T:p.H128L	Cirrhosis cryptogenic	RCV000015686
12	103237484	rs62642937	G	A	42/2/0	*PAH*	uc001tjq.1:c.C1140T:p.T380M	Hyperphenylalaninemia non-pku	RCV000000660
12	121174897	rs61732144	C	T	41/3/0	*ACADS*	uc001tza.3:c.C320T:p.R107C	Deficiency of butyryl-CoA dehydrogenase	RCV000004030

12	121175678	rs1800556	C	T	42/2/0	*ACADS*	uc001tza.3:c.C512T:p.R171W	Deficiency of butyryl-CoA dehydrogenase	RCV000004034
13	52508989	rs60986317	G	A	43/1/0	*ATP7B*	uc001vfw.2:c.C4302T:p.T1434M	Wilson's disease	RCV000029379
13	52518281	rs76151636	G	T	42/2/0	*ATP7B*	uc001vfw.2:c.C3208A:p.H1070Q	Wilson's disease	RCV000004052
13	113795286	rs61753266	G	A	43/1/0	*F10*	uc001vsx.2:c.G425A:p.E142K	Factor X deficiency	RCV000012841
14	24731434	rs41295338	G	T	42/2/0	*TGM1*	uc001wod.2:c.C126A:p.S42Y	Autosomal recessive congenital ichthyosis 1	RCV000013298
15	28228553	rs74653330	C	T	42/2/0	*OCA2*	uc001zbh.3:c.G1442A:p.A481T	Tyrosinase-positive oculocutaneous albinism	RCV000001005
15	80472526	rs11555096	C	T	43/1/0	*FAH*	uc002bfm.1:c.C1022T:p.R341W	Fumarylacetoacetase pseudodeficiency	RCV000012643
16	3293310	rs28940579	A	G	41/3/0	*MEFV*	uc002cun.1:c.T2178C:p.V726A	Familial Mediterranean fever	RCV000002649
16	8905010	rs28936415	G	A	43/1/0	*PMM2*	uc002czf.3:c.G423A:p.R141H	Carbohydrate-deficient glycoprotein syndrome type I	RCV000008145
16	17564311	rs61758388	C	A	41/3/0	*XYLT1*	uc002dfa.2:c.G344T:p.A115S	Pseudoxanthoma elasticum modifier of severity of	RCV000002643
16	23360165	rs35731153	C	G	43/1/0	*SCNN1B*	uc002dln.2:c.C246G:p.S82C	Bronchiectasis	RCV000009392
16	31105945	rs61742245	C	A	43/1/0	*VKORC1*	uc002eas.2:c.G107T:p.D36Y	Warfarin response	RCV000002296
16	31501798	rs61742739	A	G	40/4/0	*SLC5A2*	uc002ecf.3:c.A1962G:p.N654S	Familial renal glucosuria	RCV000013768
16	47549473	rs56257827	G	T	41/3/0	*PHKB*	uc002eev.3:c.G556T:p.M186I	Glycogen storage disease IXb	NA
17	3402294	rs28940279	A	C	43/1/0	*ASPA*	uc002fvq.2:c.A855C:p.E285A	Spongy degeneration of central nervous system	RCV000002723
17	7189048	rs121434581	G	A	42/2/0	*SLC2A4*	uc002gfp.2:c.G1148A:p.V383I	Diabetes mellitus type 2	RCV000017469
17	41055964	rs1801175	C	T	43/1/0	*G6PC*	uc002icb.1:c.C248T:p.R83C	Glycogen storage disease type 1A	RCV000012778
17	42328579	rs121912759	G	A	43/1/0	*SLC4A1*	uc002igf.3:c.C2604T:p.P868L	Acanthocytosis due to band 3 ht	RCV000019361
17	56356502	rs56378716	A	G	42/2/0	*MPO*	uc002ivu.1:c.T753C:p.M251T	Myeloperoxidase deficiency	RCV000003812
18	44104697	rs75949023	G	A	43/1/0	*LOXHD1*	uc010xcw.1:c.C4715T:p.R1572*	Deafness autosomal recessive 77	RCV000023981
19	7125518	rs1799816	C	T	41/3/0	*INSR*	uc002mgd.1:c.G3035A:p.V1012M	Diabetes mellitus type 2	RCV000015822
19	17953950	rs55778349	G	C	43/1/0	*JAK3*	uc002nhn.3:c.C453G:p.P151R	Severe combined immunodeficiency disease	RCV000030094
19	41354533	rs1801272	A	T	41/3/0	*CYP2A6*	uc002opl.3:c.T480A:p.L160H	Warfarin response	RCV000018482
20	43280227	rs73598374	C	T	35/8/1	*ADA*	uc002xmj.2:c.G23A:p.D8N	Adenosine deaminase 2 allozyme	RCV000002050

22	50518820	rs121908345	G	A	43/1/0	*MLC1*	uc003bjg.1:c.C275T:p.P92S	Megalencephalic leukoencephalopathy with subcortical cysts 1	RCV000004984
22	50962684	rs74315510	G	A	43/1/0	*SCO2*	uc003blz.3:c.C158T:p.Q53*	Cardioencephalomyopathy fatal infantile due to cytochrome c oxidase deficiency	RCV000006032
